# Integrin ß4 is a receptor for emerging fungal pathogens from the genera *Lomentospora* and *Scedosporium*

**DOI:** 10.1371/journal.ppat.1014107

**Published:** 2026-04-08

**Authors:** Povilas Kavaliauskas, Riley Risteen, Sondus Alkhazraji, Breanna Shirtliff, Thomas J. Walsh, Ashraf S. Ibrahim, Vincent M. Bruno

**Affiliations:** 1 Department of Microbiology and Immunology, University of Maryland School of Medicine, Baltimore, Maryland, United States of America; 2 Institute of Genome Sciences, University of Maryland School of Medicine, Baltimore, Maryland, United States of America; 3 Division of Infectious Diseases, The Lundquist Institute for Biomedical Innovation, Harbor-University of California at Los Angeles (UCLA) Medical Center, Torrance, California, United States of America; 4 Center for Innovative Therapeutics and Diagnostics, Richmond, Virginia, United States of America; 5 Department of Medicine, University of Maryland School of Medicine, Baltimore, Maryland, United States of America; 6 David Geffen School of Medicine at UCLA, Los Angeles, California, United States of America; Texas Tech University Health Sciences Center School of Medicine - Lubbock Campus: Texas Tech University Health Sciences Center School of Medicine, UNITED STATES OF AMERICA

## Abstract

*Lomentospora prolificans* is an environmental fungus that can cause life-threatening infections when airborne conidia are inhaled. Airway epithelial cells are likely to be the first host cells to interact with *L. prolificans* during pulmonary infection; however, the fungal and host factors that govern this interaction are completely unknown. Herein, we combined whole fungal cell pulldowns of surface proteins from airway epithelial cells and liquid chromatography-mass spectrometry (LC–MS) to identify host proteins that could potentially serve as host receptors for the fungus. We provide evidence that integrin β4 serves as a receptor that promotes the initial binding of *L. prolificans* to airway epithelial cells. Integrin β4 can associate with *L. prolificans* conidia that have been heat-killed or pre-treated with proteinase K suggesting that the fungal ligand is not proteinaceous. Inhibition of integrin β4 function by siRNA-mediated knockdown, or blocking with an anti-integrin β4 antibody, significantly inhibited the ability of *L. prolificans* to adhere to human airway epithelial cells. Integrin β4 can also associate with, and promote the adherence of, two closely related species of fungal pathogens, *Scedosporium apiospermum* and *Scedosporium boydii*. Overall, our study provides novel insight into the molecular mechanisms underlying the initiation of infection by *L. prolificans,* and two closely related species.

## Author summary

*Lomentospora prolificans* is recognized as a priority fungal pathogen by the World Health Organization due to its rising clinical significance to cause life-threatening infections that are highly resistant to most licensed antifungal agents. As a causative agent of severe invasive fungal diseases in both immunocompromised and immunocompetent individuals, *L. prolificans* poses considerable challenges in healthcare settings. Our study addresses crucial gaps in the understanding of the molecular interactions between *L. prolificans* and human cells. Here we identify integrin *β*4 as a mediator of *L. prolificans* adhesion to lung airway epithelial cells. Overall, our study provides novel insight into the molecular mechanisms underlying the initiation of infection by *L. prolificans,* and two closely related species.

## Introduction

*Lomentospora prolificans* is a filamentous, dematiaceous, emerging fungal pathogen responsible for infections in both immunocompromised and immunocompetent individuals [1,2]. These infections can occur at various anatomical sites, with disseminated and pulmonary infections being the most prevalent and deadly forms of the disease [2–4]. Infections caused by *L. prolificans* are highly resistant to most licensed antifungal agents with a paucity of therapeutic options [5]. The respiratory tract is the main portal of entry for disseminated infections that occur in immunocompromised patients. In immunocompetent individuals, the invasive cases often occur as a consequence of direct traumatic inoculation into musculoskeletal tissue [6,7]. In the respiratory tract, airway epithelial cells line the mucosa and act as a physical barrier against inhaled fungal particles. These cells also produce and secrete antimicrobial peptides that can serve as a biochemical barrier to fungal invasion as well as recruit innate immune cells to clear the infection via their ability to produce cytokines and chemokines [8]. Most studies of *L prolificans* have examined interaction with neutrophils, monocytes, or macrophages [9]. However, with the exception of one study [10], the interaction between *L. prolificans* and lung epithelial cells has not been thoroughly explored. Herein, we examine the interaction between *L. prolificans* and a normal, Tert-immortalized, small human airway epithelial cell line (HSAEC1-KT).

### Results

### Integrin *β*4 from airway epithelial cells can bind to *L. prolificans*

**Fig 1:**
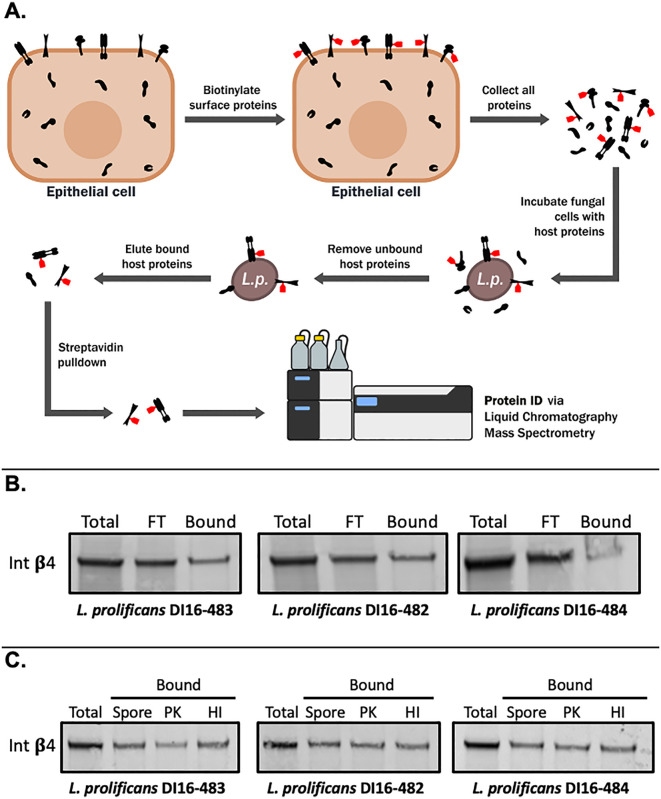
Integrin *β*4 associates with *L. prolificans.* (A) Schematic of methods used to identify host proteins that can associate with *L. prolificans* conidia. (B) *L. prolificans*-bound HSAEC1-KT surface proteins were separated by SDS-PAGE and immunoblotted using an anti-Integrin β4 antibody. Total, total cell surface lysates; FT, flow-through; Bound, eluted proteins that bound to the fungal conidia. (C) HSAEC1-KT surface proteins that bound to untreated (Spore), Proteinase K-treated (PK) or heat-inactivated (HI) *L. prolificans* conidia were separated by SDS-PAGE and immunoblotted using an anti-Integrin β4 antibody.

Pulmonary infection is initiated when airborne conidia are inhaled, or aspirated into the lungs, and then deposited into the airways. The initial adhesion of fungal pathogens to airway epithelial cells plays a crucial role in subsequent steps of pathogenesis [11]. Therefore, we set out to identify host cell surface proteins that can physically interact with *L. prolificans* isolate DI16–483 by performing whole fungal cell pulldowns with biotinylated HSAEC1-KT surface proteins, followed by proteomic analysis using liquid chromatography-mass spectrometry (LC–MS, Fig 1A). Since this isolate of *L. prolificans* has only been studied in the context of an *in vitro* antifungal susceptibility assay [12], we first confirmed its ability to cause disease using an *in vivo* neutropenic murine pulmonary infection model [12]. In the murine pulmonary infection model, *L. prolificans* (DI16–483) is able to establish infection in the lung and cause significant lethality with a median survival time of five days post-inoculation (S1A, S1B Fig).

Since our goal was to identify host cell surface proteins that can bind to *L. prolificans*, we used three criteria to determine if a host protein is a potential receptor for *L. prolificans*. First, we required that our LC-MS analysis identify at least 10 peptides of a given protein. Second, the protein must be listed as a “high confidence” hit in the Cell Surface Protein Atlas [13]. Third, the protein must be annotated as a “Receptor” in the UniProt database (https://www.uniprot.org). Our analysis identified five potential receptors for *L. prolificans*: Plexin-B2, Transferrin receptor protein 1 (TFRC), Solute Carrier Family 3 Member 2 (SLC3A2), Receptor-type tyrosine-protein phosphatase F (PTPRF), and integrin β4 (S1 Table). None of these proteins have been reported to be involved in direct host cell interactions with any fungal pathogen. As other members of the integrin protein family are known to serve as receptors for fungal pathogens [14–16] and integrin β4 is known to be expressed in the lungs of both humans and mice [17–19], we selected this protein for further investigation.

In order to verify that integrin β4 from airway epithelial cells can bind to *L. prolificans* conidia, we used a commercially available anti-integrin β4 antibody to probe immunoblots containing HSAEC1-KT membrane proteins that were enriched for binding to conidia. This antibody recognized a conidium bound 210-kDa band derived from HSAEC1-KT cells (Fig 1B) and A549 cells (human type II alveolar cells) (S1C Fig). We also observed an association between integrin β4, derived from HSAEC1-KT cells and conidia of two additional isolates of *L. prolificans* (DI16–482 and DI16–484) (Fig 1B). We did not recover integrin β4 protein in negative control, empty tube samples in which the fungus was left out of the reaction (S1D Fig). Heat killing or pre-treatment of conidia with Proteinase K did not inhibit association with integrin ß4 (Fig 1C). These results indicate that integrin β4 binding is not strain-specific and that integrin β4 is most likely not binding to a protein on the conidial surface of *L. prolificans*. To address the possibility that integrin β4 is non-specifically sticking to cell wall material, we performed pulldowns with isolates from two gram-positive bacteria species (*Staphylococcus aureus* and *Enterococcus faecalis*) and two gram-negative species (*Klebsiella pneumoniae* and *Pseudomonas aeruginosa*). We could not detect integrin β4 binding to any of these isolates (S1E Fig).

### Integrin *β*4 promotes *L. prolificans* adherence to airway epithelial cells

**Fig 2:**
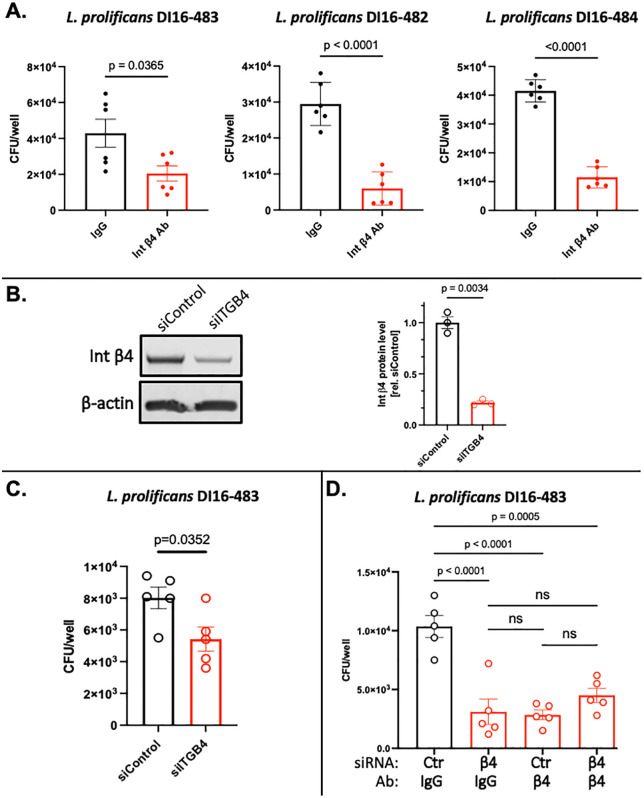
Integrin *β*4 promotes *L. prolificans* adherence to airway epithelial cells. (A) Adherence of three different isolates of *L. prolificans* to HSAEC1-KT cells 3 h post-infection following pre-treatment with an anti-Integrin β4 antibody or an IgG control antibody. (B, left panel) Representative immunoblot demonstrating depletion of Integrin β4 by siRNA knockdown. HSAEC1-KT cells were exposed to siRNAs for 48 h prior to infection, which was allowed to progress for 3 h. Experiment was performed three times with similar results. (B, right panel) Densitometric analysis of the immunoblot. (C) Adherence of *L. prolificans* isolate DI16-483 to HSAEC1-KT cell 3 h post-infection following depletion of Integrin β4 by siRNA knockdown demonstrated by colony forming unit (CFU) assay. (D) Adherence of *L. prolificans* isolates DI16-483 to HSAEC1-KT cell 3 h post-infection following depletion of Integrin β4 by siRNA knockdown, antibody blocking, or a both. All values represent the mean ± SEM.

We next sought to determine whether blocking the function of integrin β4 would prevent adherence of *L. prolificans* to airway epithelial cells. HSAEC1-KT cells were pre-treated with an anti-integrin β4 antibody or an isotype control, then infected with *L. prolificans* for 3 hours*.* Compared to pre-treatment with an isotype-matched control IgG, the anti-integrin β4 antibody reduced the adherence of all three *L. prolificans* isolates by more than 50% (Fig 2A). Furthermore, the decrease in fungal adherence correlated with the concentration of anti-integrin β4 antibody added to the cells (S2 Fig).

We also examined whether knocking-down the expression of integrin β4 in epithelial cells would reduce fungal adhesion. Treatment of HSAEC1-KT cells with integrin β4-directed siRNA resulted in a ~ 75% inhibition of integrin β4 protein expression (Fig 2B) and a significant reduction in the ability *L. prolificans* to adhere to the host cells (Fig 2C). In a complementary approach, we examined the effects of combining antibody blockade and siRNA inhibition on fungal adhesion. As observed in other experiments (Fig 2A-C), pre-treatment with anti-integrin β4 antibody or siRNA alone each significantly reduced fungal adhesion (Fig 2D). Notably, treating the host cells with both the anti-integrin β4 antibody and siRNA resulted in equivalent levels of inhibition as each individual treatment (Fig 2D), confirming the specificity of the antibody-based inhibition in our experiments. Together, these results suggest that integrin β4 promotes adherence of *L. prolificans* to small airway epithelial cells.

### Integrin *β*4 binds to and promotes adherence of *S. apiospermum* and *S. boydii* to airway epithelial cells

**Fig 3:**
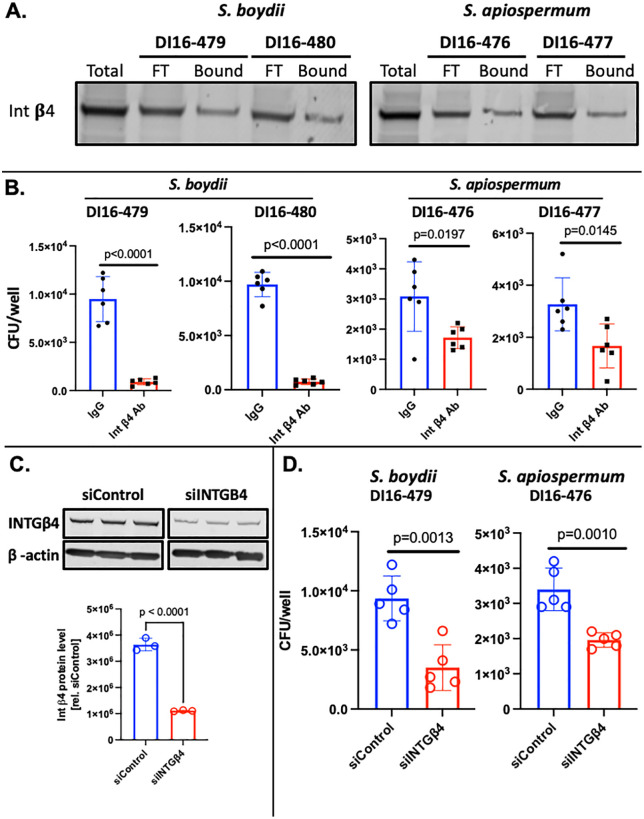
Integrin *β*4 associates with *S. boydii and S. apiospermum* and promotes adherence to airway epithelial cells. (A) *S. boydii- or S. apiospermum*-bound HSAEC1-KT surface proteins were separated by SDS-PAGE and immunoblotted using an anti-Integrin β4 antibody. Total, total cell surface lysates; FT, flow-through; Bound, eluted proteins that bound to the fungal conidia. (B) Adherence *S. boydii* and *S. apiospermum* isolates to HSAEC1-KT cells 3 h post-infection following pre-treatment with an anti-Integrin β4 antibody or an IgG control antibody. (C, top panel) Immunoblot representing depletion of Integrin β4 by siRNA knockdown. HSAEC1-KT cells were exposed to siRNAs for 48 h prior to infection, which was allowed to progress for 3 h. Each lanes represents a biological replicate. (B, bottom panel) Densitometric analysis of the immunoblot. (D) Adherence of *S. boydii* isolate DI-16-479 and *S. apiospermum* isolate DI16-476 to HSAEC1-KT cell 3 h post-infection following depletion of Integrin β4 by siRNA knockdown. All values represent the mean ± SEM.

We next sought to determine if integrin β4 can also bind to pathogenic isolates of two closely related species of emerging fungal pathogens, *Scedosporium apiospermum* and *Scedosporium boydii*. From the time of its initial discovery until 2014, *L. prolificans* was considered a *Scedosporium* species [20,21]. Immunoblotting of HSAEC1-KT membrane proteins that were enriched for binding to two different *S. apiospermum* isolates (DI16–476 and DI16–477) and 2 different *S. boydii* isolates (DI16–479 and DI16–480) demonstrated binding to integrin β4 to all four isolates (Fig 3A). The adherence of each of these isolates to HSAEC1-KT cells was significantly reduced by blocking with an anti-integrin β4 antibody (Fig 3B). In a complementary set of experiments, treatment of HSAEC1-KT cells with integrin β4-directed siRNA resulted in a ~ 70% inhibition of integrin β4 protein expression (Fig 3C) and a significant reduction in the ability *S. boydii* and *S. apiospermum* to adhere to the host cells (Fig 3D). These data suggest that the ability to associate with integrin β4 and promote fungal adherence is not a property that is unique to *L. prolificans* but also applies to at least two additional closely related species.

## Discussion

In this work, we investigated the *in vitro* interaction between *L. prolificans* and human airway epithelial cells to identify host proteins that promote this interaction. The most salient findings of our study are [1] integrin β4 can bind to conidia of *L. prolificans;* [2] this binding can occur with both heat-killed and protease K treated conidia; [3] pre-treatment of airway epithelial cells with an anti-integrin β4 antibody reduces fungal adhesion; and [4] integrin β4 also binds to the conidia of, and promotes the adhesion of, *S. apiospermum* and *S. boydii,* two species that are closely related to *L. prolificans.*

Integrin *β*4 forms a complex with integrin *α*6 to serve as a receptor for laminin and as a cellular adhesion molecule [22–24]. Signaling from the integrin *α*6*β*4 complex regulates various cellular processes, including cell migration, survival, and angiogenesis [23,24]. Integrin *β*4, alone or in complex with integrin *α*6, can also mediate host cell attachment and infection of Zika virus [25]. Notably, our LC-MS analysis did not detect any integrin *α*6 peptides among our enriched surface proteins and our analysis suggests that integrin β4 binding to conidia likely does not involve a fungal protein (Fig 1C). Furthermore, the interaction between *L. prolificans* and integrin β4 could not be inhibited by the addition of excess laminin to the binding reaction (S1F Fig). These observations suggest that integrin β4 binds to conidia in a manner that is independent of both integrin *α*6 and the integrin β4 laminin-binding domain.

The ability of integrins to function as fungal adhesion molecules has been documented among evolutionarily diverse fungi. The CalA protein of *Aspergillus fumigatus* binds to host integrin *α*5*β*1 expressed on pulmonary epithelial cells to facilitate host cell invasion [16]. Similarly, *Candida albicans* hyphae can bind to integrin *α*X*β*2 in a *β*-glucan-sensitive manner [15]. Furthermore, Mucorales fungi bind to integrin α3β1 on lung epithelial cells through the action of the fungal spore coat protein encoded by CotH7 [14]. Notably, a role for integrin *β*4 in fungal-host interactions has not been previously described.

Our siRNA and antibody-mediated receptor blocking studies (Fig 2) suggest a role for integrin *β*4 in the initial interaction between *L. prolificans* airway epithelial cells, specifically as a fungal adhesion receptor. At the moment, the fungal ligand(s) that interacts with integrin β4 remains unknown. Our current data suggest that the ligand of *L. prolificans*, *S. boydii*, and *S. apiospermum* for integrin β4 is a non-protein molecule. Additional experiments are required to (i) identify the fungal ligand(s), (ii) identify other host factors that might be involved in the integrin β4-mediated binding, (iii) characterize the fungal associated downstream signaling events of integrin β4-mediated adhesion, and (iv) understand the broader role (*e.g.,* fungal invasion and host immune response) of integrin β4 in the pulmonary host defense and development of fungal infections caused by isolates of the genera *Lomentospora* and *Scedosporium*.

## Materials and methods

Details regarding Materials and Methods and key reagents and resources can be found in the **Supplementary Text**.

## Supporting information

S1 Fig (A) Survival of neutropenic mice (10 per group) infected intratracheally with conidia of *L. prolificans* isolate DI16–483. (B) Representative images of GMS-stained lung tissue from two different mice infected with L. *prolificans* isolate DI16–483. (C) *L. prolificans*-bound A549 surface proteins were separated by SDS-PAGE and immunoblotted using an anti-Integrin β4 antibody. Total, total cell surface lysates; FT, flow-through; Bound, eluted proteins that bound to the fungal conidia. (D) Representative blot of several experiments performed with empty tube (no fungus) control. (E) HSAEC1-KT surface proteins that bound to conidia of *L. prolificans* isolate DI16–483, or various bacterial isolates, were separated by SDS-PAGE and immunoblotted using an anti-Integrin β4 antibody. TL, total lysates; FT, flow-through; Bound, eluted proteins that bound to the microorganism; ET, empty tube control; L.p, *L. prolificans* isolate DI16–483; *S.a., Staphylococcus aureus; E.f., Enterococcus faecalis; K.p., Klebsiella pneumoniae; P.a., Pseudomonas aeruginosa.* (F) HSAEC1-KT surface proteins that bound to conidia of *L. prolificans* isolate DI16–483 in the presence of varying concentrations of purified laminin were separated by SDS-PAGE and immunoblotted using an anti-Integrin β4 antibody. Total, total cell surface lysates; FT, flow-through; Bound, eluted proteins that bound to the fungal conidia.
(TIFF)

S2 Fig (A) Adherence of *L. prolificans* isolate DI16–483 to HSAEC1-KT cells 3 h post-infection following pre-treatment with three different concentrations of an anti-Integrin β4 antibody or an IgG control antibody. All values represent the mean ± SEM. (B) Scatter plot of data in Panel A demonstrating that the relative adherence decreases with increasing antibody concentration.
(TIFF)

S1 Table List of protein hits from the LC-MS data. (XLSX)

S1 Text This document contains a detailed “Materials and Methods” section describing the experiments and analyses performed. (PDF)
