# Supplementary figures and images for: Integrin ß4 is a receptor for emerging fungal pathogens from the genera Lomentospora and Scedosporium
Source: PLoS Pathog. 2026 Apr 8;22(4):e1014107. doi: 10.1371/journal.ppat.1014107 (PMC13061235; doi:10.1371/journal.ppat.1014107)

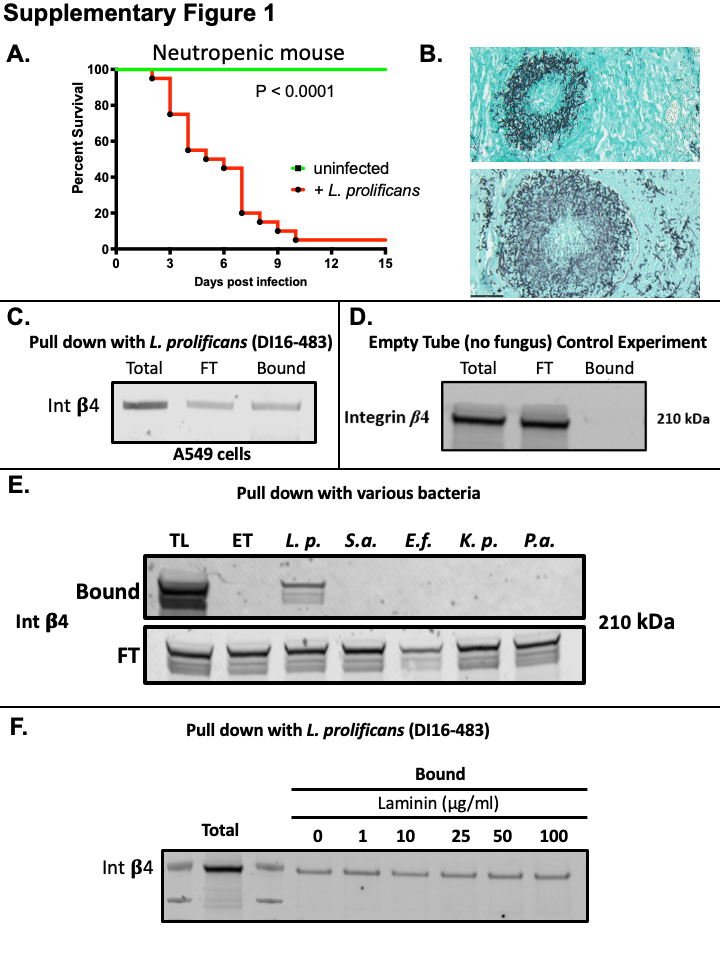

Supplement: S1 Fig — (A) Survival of neutropenic mice (10 per group) infected intratracheally with conidia of L. prolificans isolate DI16–483. (B) Representative images of GMS-stained lung tissue from two different mice infected with L. prolificans isolate DI16–483. (C) L. prolificans-bound A549 surface proteins were separated by SDS-PAGE and immunoblotted using an anti-Integrin β4 antibody. Total, total cell surface lysates; FT, flow-through; Bound, eluted proteins that bound to the fungal conidia. (D) Representative blot of several experiments performed with empty tube (no fungus) control. (E) HSAEC1-KT surface proteins that bound to conidia of L. prolificans isolate DI16–483, or various bacterial isolates, were separated by SDS-PAGE and immunoblotted using an anti-Integrin β4 antibody. TL, total lysates; FT, flow-through; Bound, eluted proteins that bound to the microorganism; ET, empty tube control; L.p, L. prolificans isolate DI16–483; S.a., Staphylococcus aureus; E.f., Enterococcus faecalis; K.p., Klebsiella pneumoniae; P.a., Pseudomonas aeruginosa. (F) HSAEC1-KT surface proteins that bound to conidia of L. prolificans isolate DI16–483 in the presence of varying concentrations of purified laminin were separated by SDS-PAGE and immunoblotted using an anti-Integrin β4 antibody. Total, total cell surface lysates; FT, flow-through; Bound, eluted proteins that bound to the fungal conidia. (TIFF) [file ppat.1014107.s001.tiff]

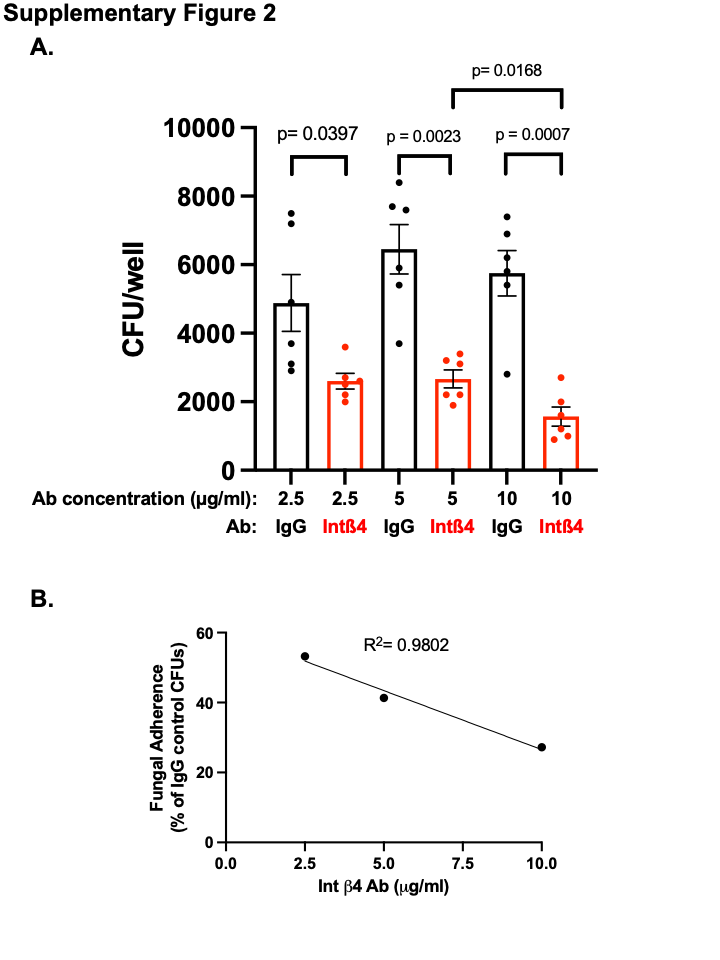

Supplement: S2 Fig — (A) Adherence of L. prolificans isolate DI16–483 to HSAEC1-KT cells 3 h post-infection following pre-treatment with three different concentrations of an anti-Integrin β4 antibody or an IgG control antibody. All values represent the mean ± SEM. (B) Scatter plot of data in Panel A demonstrating that the relative adherence decreases with increasing antibody concentration. (TIFF) [file ppat.1014107.s002.tiff]
