# Supplementary material for: Integrin ß4 is a receptor for emerging fungal pathogens from the genera Lomentospora and Scedosporium
Source: PLoS Pathog. 2026 Apr 8;22(4):e1014107. doi: 10.1371/journal.ppat.1014107 (PMC13061235; doi:10.1371/journal.ppat.1014107)
Supplement: S1 Text — (PDF) [file ppat.1014107.s004.pdf]

Supplementary Text

MATERIALS AND METHODS

Strains and culture conditions. The clinical isolates of *Lomentospora prolificans* (DI16-482, DI16-483, DI16-484), *Scedosporium boydii* (DI16-479, DI16-480) and *Scedosporium apiospermum* (DI16-476, DI16-478) were obtained from the Fungus Testing Laboratory at the University of Texas Health Sciences Center at San Antonio (UTHSCSA). Fungi were cultured on peptone-dextrose agar (PDA) plates for 7 days at 37°C prior to experiments. For infections, conidia were harvested in endotoxin-free Dulbecco's phosphate-buffered saline (DPBS) containing 0.5% Tween 80, subsequently washed with endotoxin-free DPBS, and enumerated using a hemocytometer to prepare the final inoculum. Prior to *in vitro* infections, enumerated conidia were suspended in Small Airway Epithelial Cell Growth Medium Basal Medium (SAGM; Lonza, Catalog #:CC-3118) and incubated at 37°C for 3 hours to allow for swelling. The bacterial isolates *Staphylococcus aureus* (BES 77), *Enterococcus faecalis* (CSF), *Klebsiella. pneumoniae* (TBE 786) and *Pseudomonas aeruginosa* (PAO1) were generated by Dr. Robert Enrst (University of Maryland School of Dentisry).

Murine model of lomentosporiosis. Male ICR mice (20 to 25 g [from Envigo]) were immunosuppressed by cyclophosphamide (200 mg/kg administered intraperitoneally [i.p.]) and cortisone acetate (500 mg/kg administered subcutaneously) given on days -2, and +3 relative to infection. This treatment resulted in 16 days of pancytopenia. To

control for bacterial infection, immunosuppressed mice received 50 mg/liter enrofloxacin (Baytril; Bayer, Leverkusen, Germany) *ad libitum* on day -3 through day 0, after which the enrofloxacin was replaced with daily ceftazidime (5 mg/mouse) treatment administered subcutaneously through day 8 relative to infection. Mice were infected with 4.3 x 10^4 conidia of *L. prolificans* DI16-683 in 25 µl PBS given intratracheally. Survival of mice served as the primary endpoint, with moribund mice humanely euthanized. All animal studies were approved by the Institutional Animal Care and Use Committee (IACUC) of the Los Angeles Biomedical Research Institute at Harbor-UCLA Medical Center according to the NIH guidelines for animal housing and care (approval reference no. 21125).

Preparation of host cell surface proteins and pulldown experiments. HSAEC1-KT or A549 cells were seeded into 150 mm Petri dishes and allowed to incubate for 7 days until reaching full confluency. The cells were then washed three times with ice-cold Dulbecco's phosphate-buffered saline (DPBS) supplemented with 1 mM phenylmethylsulfonyl fluoride (PMSF). To label cell surface proteins, cell-impermeable Sulfo-NHS-SS-biotin (BroadPharm, Catalog#BP-22634) was diluted in ice-cold PBS (pH 8.0) to a final concentration of 0.5 mg/ml and added to the HSAEC1-KT cells, followed by incubation on ice for 1 hour. After incubation, the biotinylation reagent was aspirated, and cells were rinsed three times with 0.1 M glycine in PBS to quench any unreacted biotinylation reagent. Subsequently, cells were washed three times with ice-cold DPBS, scraped, and suspended in membrane protein lysis buffer (comprising 5.6% octyl beta-glucoside, DPBS, 1 mM PMSF, and a protease inhibitor cocktail), followed by incubation

on ice for 30 minutes and sonication (45% amplitude, 10s cycles). The biotinylated cell lysates were then centrifuged at 10,000 × g for 30 minutes at 4°C. Protein concentrations were determined using a BCA assay (Pierce, ThermoFisher), and biotinylated protein lysates were utilized for mass spectrometry proteomic analysis experiments. Non-biotinylated HSAEC1-KT proteins intended for pulldown experiments and Western blot validation were prepared as described above, with the exclusion of biotinylation steps. Pulldown assays were conducted as previously described (24)with modifications. Approximately 5 x 10^8 conidia were utilized for the pulldown experiments. Swollen conidia were washed three times with ice-cold Dulbecco's phosphate-buffered saline (DPBS) supplemented with 1 mM PMSF and suspended in DPBS. For proteomic mass spectrometry experiments, 1 mg of biotinylated proteins were added to the conidia. For Western blotting experiments, 2.5 mg of non-biotinylated HSAEC1-KT proteins were added. The pulldown was conducted overnight at 4°C with constant mixing. Subsequently, the conidia were washed three times using two volumes of ice-cold DPBS supplemented with 1 mM PMSF. After washing, the conidia were transferred to a fresh tube, and proteins were eluted using elution buffer (composed of 6M urea, 5.6% octyl beta-glucoside, DPBS, and 1 mM PMSF) followed by sonication (3 cycles, 45% amplitude, 10s each). For heat inactivation, conidia were incubated for 1 hour at 70 °C. In order to ensure that the conidia were dead, 10 µl of the heated conidia were plated onto PDA plates and grown at 37 °C for 5 days. For Proteinase K treatment, 5 x 10^8 conidia were suspended in a solution of 100 µg/ml of Proteinase K (Cat.# 70663, Sigma-Aldrich) for 1 hour at 37 °C. After the Proteinase K digestion, the conidia were

washed two times with DPBS, 1 mM PMSF, 1X protease inhibitor cocktail (Cat.# P2714, Sigma-Aldrich) to quench the Proteinase K activity.

Proteomic analysis

For proteomic LC-MS analysis and host protein identification, biotinylated eluted proteins obtained from pulldown experiments were dialyzed overnight in Tris-buffered saline (TBS). Subsequently, the biotinylated HSAEC1-KT surface proteins were enriched using streptavidin purification (Pierce, ThermoFisher Scientific). Proteins bound to streptavidin beads were eluted by heating the beads in reducing LDS sample loading buffer (Pierce, ThermoFisher Scientific). Following the enrichment of biotinylated proteins, the samples were digested overnight with trypsin at 37 °C. The digests were desalted by C18 Stage-tip columns and analyzed using a Thermo Fisher Scientific EASY-nLC 1200 coupled on-line to a Fusion Lumos mass spectrometer (Thermo Fisher Scientific) operated in a data independent acquisition (DIA) mode. The data were searched against the human Uniprot database using DIA-NN v1.8 and filtered for 1% false discovery rate for both protein and peptide identifications. LC-MS analysis was conducted at the Metabolomics and Proteomics Core Facility at Weill Cornell Medicine of Cornell University. The raw LC-MS data is publicly available at 10.5281/zenodo.18745901.

Culture and siRNA transfection of Human cell lines

Human small airway epithelial cells (HSAEC1-KT; CRL-4050) were purchased from ATCC and cultured in flasks containing Small Airway Epithelial Cell Growth Medium

(SAGM), supplemented with PenStrep (Gibco, ThermoFisher) at 37°C in a humidified atmosphere with 5% CO_2. The cells were replenished with fresh medium every 2 to 3 days and passaged once weekly. For transfection, cells were plated at a seeding density of 0.25 × 10^6 cells/ml to reach a confluency of 80-90% on the day prior to transfection. Xfect (Cat# 631450, Takara Clontech) was used to transfect cells with 100 pmol siRNAs or with control siRNA. The growth medium was replaced 4-hr post-transfection. Cells were harvested 48 hours post transfection for downstream experimental analysis.

The A549 type II pneumocyte cell line (American Type Culture Collection) was grown in Dulbecco's Modified Eagle Medium (DMEM) (Cat#11965092, Gibco)containing 10% fetal bovine serum (Gemini Bio-Products) and 1% streptomycin and penicillin (Irvine Scientific) in 5% CO_2 at 37°C.

Western blotting

The eluted proteins obtained from pulldown experiments using non-biotinylated HSAEC1-KT proteins were separated by NuPAGE™ 4-12% Bis-Tris gels (Thermo Fisher, #WG1402BOX). Following electrophoresis, proteins were transferred to polyvinylidene fluoride (PVDF) membranes. The membranes were blocked for 1 hour in Intercept Blocking Buffer (LI-COR, #927-60001), then probed with a 1:1,000 dilution of rabbit integrin β4 antibody (Cell Signaling Technologies; D8P6C, mAb #14803) and incubated overnight at 4°C. To confirm HSAEC1-KT biotinylation, membranes were probed with rabbit anti-biotin antibody (Cell Signalling Technologies, # mAb #5597). Subsequently, the membranes were washed three times with Tris-buffered saline with

Tween 20 (TBS-T) and incubated for 1 hour with a 1:15,000 dilution of secondary antibodies (LI-COR, #926-68072). Following another three washes with TBS-T, bands were visualized using an Odyssey CLx imaging system (LI-COR).

Adherence of *L. prolificans* to epithelial cells. Six days prior to the experiments, HSAEC1-KT cells were seeded in 12-well plates and cultured until they reached full confluency, approximately 1.4 x 10^6 cells per well. On the day of infection, the media was aspirated, and cells were rinsed with Dulbecco's phosphate-buffered saline (DPBS). Fresh medium containing rat anti-integrin β4 antibody (Invitrogen, Catalog #: 14-1049-82) at a dilution of 1:100 was added, followed by incubation for 1 hour at 37°C. Rat IgG was used as an isotype control (Invitrogen, Catalog #: 02-9602). Unless otherwise noted, the final concentration of the antibodies on the cells was 5 μg/ml. After incubation with the antibodies and isotype control, the medium was replaced with fresh medium containing antibodies, and the cells were infected with swollen *L. prolificans* at a multiplicity of infection (MOI) of 1:5. The infection was synchronized by centrifugation for 1 minute at 1000 x g, and infection was allowed to proceed for 3 hours. Following infection, the media was removed, and cells were washed three times with DPBS. The cell monolayer was then lysed using 0.5% Triton X-100 in deionized water and scraped using a cell scraper. The cell lysates were serially diluted in PBS and plated on peptone-dextrose agar (PDA). Plates were then incubated at 37°C for 7 days, and colony-forming units per well (CFU/well) were calculated.

Statistics and reproducibility. No statistical tests were utilized to pre-determine sample size. As it is the minimum number of replicates required for inferential analysis, at least three biological replicates were utilized for all experiments. No data were excluded from analyses, the experiments were not randomized, and investigators were not blinded to allocation during experiments of outcomes assessment. Statistical analyses were performed using GraphPad Prism 10.2.0 for Windows (GraphPad Software, SanDiego, CA, USA). Two-tailed Student t-tests were used for statistical analyses in Figures 2 and 3. Survival curves were analyzed with the Log-Rank (Mantel-Cox) test.
